# Irreversible effects of youthful choices in aged adults

**DOI:** 10.1371/journal.pgen.1008218

**Published:** 2019-07-11

**Authors:** Alana M. O’Reilly

**Affiliations:** Molecular Therapeutics Program, Fox Chase Cancer Center, Philadelphia, Pennsylvania, United States of America; College de France CNRS, FRANCE

The choices we make can have long-lasting effects on our health. Repetitive stress; diet; use of cigarettes, drugs, or alcohol; and work-related exposure to environmental toxins often exhibit no immediate effects. Symptoms can appear later on, sometimes many decades later, making it challenging to assign specific blame to behaviors that occurred in the past.

Most impactful are behaviors that cause irreversible changes to impair tissue function. The paper by Yi-Chieh Chang and colleagues [1] describes a novel mechanism explaining how behavioral choices made as part of normal life impact long-term gamete production. The work builds on previous studies showing that continuous mating in several organisms results in sterility [2]. The authors find that fertility arrest in continuously mated *Drosophila melanogaster* males is caused by an early spermatogenesis arrest. Instead of proceeding through development normally, precursor germline and somatic cells accumulate as immature progenitors, causing a nearly complete block in spermatogenesis. Through careful investigation, the authors discovered that continuous mating causes developmental arrest indirectly, via induction of inflammation signals in the muscle sheath that surrounds the testes. Thus, it appears that muscle exhaustion or mating-associated muscle damage impacts cell fate determination nonautonomously.

Gamete quality is paramount for maintenance of species, with multiple mechanisms contributing to age-associated fertility arrest [3]. The best studied of these is loss of germline stem cells (GSCs). GSCs in the fly testes reside near a group of somatic cells known as the “hub,” with reciprocal signals between the two cell types maintaining a pool of GSCs in direct contact with hub cells [4]. Molecular changes that impact the function of GSCs and hub cells have been implicated in aging-associated GSC loss, with defects in either cell type leading to fertility arrest [5–9]. A second population of stem cells, the cyst stem cells (CySCs), associate with the hub and intermingle with GSCs. Signals and interactions between GSCs, CySCs, and hub cells are critical for the asymmetric divisions that maintain pools of self-renewing stem cells and generate the daughter cells that differentiate to produce functional sperm [4]. Shifts in the delicate balance between self-renewal and differentiation have significant impact on fertility, with accumulation of undifferentiated cells resulting in sterility [4].

Chang and colleagues focus on the effects of aging on the non-self-renewing daughters of GSCs and CySCs [1]. Reciprocal signaling between a GSC-derived gonialblast and two tightly associated daughter cells of CySCs (cyst cells) controls proliferation, survival, and differentiation during early spermatogenesis [10]. The authors make the striking finding that continuously mated males accumulate undifferentiated cyst cells with characteristics of CySCs, often at distances far away from the normal location of CySCs at the hub. Although unmated males exhibit age-associated changes in the testes and other tissues, no accumulation of poorly differentiated cyst cells is seen, supporting the notion that mating directly impacts the ability of cyst cells to properly differentiate. Immature germ cells accumulate in parallel to poorly differentiated cyst cells, an expected result given the tightly controlled reciprocity in signaling between the two cell types. Incredibly, accumulation of poorly differentiated cyst cells depends on the degree of mating. Males paired with single females were less impacted than those paired with six or more females, demonstrating that the rate of mating is proportional to impaired differentiation.

Numerous studies have demonstrated the critical role of transforming growth factor beta (TGFβ) signaling in promoting GSC and CySC self-renewal by blocking differentiation [4]. The authors found that reducing expression of TGFβ signals in aged, mated males abrogates accumulation of undifferentiated germ cells, supporting the idea that increased production of the TGFβ ligand *dpp* by poorly differentiated cyst cells blocks germ cell differentiation. However, reducing *dpp* expression has no effect on accumulation of poorly differentiated cyst cells, indicating that a previously undiscovered mechanism must regulate cyst cell differentiation.

Using clever deductive reasoning, Chang and colleagues determined that c-Jun N-terminal kinase (JNK) activity within cyst cells blocks their differentiation. They narrowed down the source of the JNK activating ligand to the muscle sheath surrounding the testes, finding that the tumor necrosis factor (TNF) ligand Eiger is both necessary and sufficient to inhibit cyst cell differentiation via JNK activation. The data support the model that excess muscle use leads to increasing Eiger production with age, with receipt of the signal by the TNF receptor Grindelwald on the cyst cell surface triggering JNK activity and the resulting differentiation block.

For years, scientists have noted the inverse impact of reproduction on fertility. This study defines a novel mechanism in which inflammatory signals in tissue-associated muscle prevent gamete production, resulting in fertility arrest. The results are congruous with the disposable soma theory, in which the germline is protected at the expense of the soma [11]. In this case, damage to the somatic muscle increases with age, correlating with spermatogenesis arrest via this interesting and novel mechanism. However, the work suggests that aging symptoms are not passive, as aged, unmated males exhibit no signs of muscle inflammation or differentiation defects. Thus, the soma accumulates damage due to specific behavior, rather than time alone, more consistent with an adaptive aging model [11].

Why does this mechanism exist? One idea is that fertility arrest occurs because of increasing genetic mutations over time, resulting in gamete defects and unsuccessful reproduction. However, Chang and colleagues show that aged, never-mated males produce differentiated sperm and can productively mate to produce progeny. This calls into question the idea that passive mutation accumulation over time is the key mechanism driving fertility arrest. Perhaps TNF produced by damaged muscle induces mutations in developing germ cells. Eiger is expressed throughout the entire muscle sheath surrounding the testes in continuously mated males [1], suggesting a potential negative impact on germ cells throughout development. Mechanistic compensation may have evolved to arrest cyst differentiation utilizing the same TNF signal, preventing development of defective germ cells. Alternatively, muscle damage might be associated with increased vulnerability to infectious agents, making necessary an adaptive mechanism to protect the germline. Understanding the reasoning behind this exciting new mechanism may provide critical insight into the evolutionary mechanisms that determine fertile life span. More generally, understanding how frequent, but normal, muscle use induces an inflammatory response with major impact on neighboring tissues could advance our understanding of how aging symptoms develop nonautonomously. An exciting possibility is that this mechanism will be widely conserved in reproductive and other tissues, with muscle damage nonautonomously inducing irreversible cellular changes that drive aging.
